# Differences in Microbial Community Structure Determine the Functional Specialization of Gut Segments of *Ligia exotica*

**DOI:** 10.3390/microorganisms13040808

**Published:** 2025-04-02

**Authors:** Zhao-Zhe Xin, Ke Ma, Yu-Zan Che, Ji-Lei Dong, Ya-Li Xu, Xin-Tong Zhang, Xi-Ye Li, Jin-Yong Zhang

**Affiliations:** Laboratory of Aquatic Parasitology, School of Marine Science and Engineering, Qingdao Agricultural University, Qingdao 266237, China

**Keywords:** *ligia exotica*, 16S rRNA gene sequencing, metagenome, metabolome, proteome, gut transit

## Abstract

*Ligia* feed on seashore algae and remove organic debris from the coastal zone, thereby playing an important role in the intertidal ecosystem. Nevertheless, the specific roles of distinct gut segments in the gut transit remain unclear. We collected and identified *Ligia exotica* specimens in the coast of Aoshanwei, Qingdao, Shandong Province, and analyzed their foreguts and hindguts for 16S rRNA, metagenomics, metabolomics, and proteomics. The concentrations of common metabolites, NO_3_^−^-N and NH_4_^+^-N, and the contents of C and N were measured. The gut transit decreased the abundances of the dominant phyla Cyanobacteria but increased Proteobacteria, Firmicutes, and Actinobacteria, and Planctomycetes and Bacteroidetes remained relatively constant. The foregut gut microbiota is involved in the carbohydrates and amino acids metabolism, as well as the decomposition of polysaccharides. The hindgut gut microbiota performs a variety of functions, including carbohydrate and amino acid metabolism, fermentation, cell motility, intracellular transport, secretion, and vesicular translocation, and the decomposition of polysaccharides, disaccharides, and oligosaccharides. The results of omics analyses and molecular experiments demonstrated that the metabolic processes involving amino acids and carbohydrates are more active in the foregut, whereas the fermentation, absorption, and assimilation processes are more active in the hindgut. Taken together, the differences in microbial community structure determine the functional specialization of different gut segments, i.e., the foregut appears to be the primary site for digesting food, while the hindgut further processes and absorbs nutrients and then excretes them.

## 1. Introduction

The wharf roach (*Ligia*), also referred to rock lice or sea slaters, is classified under the Arthropoda, Malacostraca, Isopoda, and Ligiidae [1,2]. The majority of *Ligia* species are found in the intertidal zone, inhabiting cliffs and rocky shores, as well as artificial structures such as dams, ports, and docks [3]. The inability to survive in water for extended periods, as well as the inability to leave the water environment entirely, precludes the possibility of long-distance migration, thereby limiting the species’ dispersal potential. *Ligia* exemplifies a transitional form of marine isopods, representing a significant evolutionary transition from marine to terrestrial environments. This unique biological specimen holds substantial research value in the field of isopod biology [4,5]. *Ligia* are omnivores, favoring plant debris and also ingesting some sand and animal food [6,7]. These organisms have been observed consuming seashore algae and facilitating the removal of organic debris from the coastal zone, thereby accelerating the chemical cycle of the Earth’s materials. *Ligia* is thought to play an active role in the cycling of nutrients and the flow of energy in the nearshore environment, and it supports biodiversity in the coastal zone [8]. Currently, approximately 43 species are included in the genus *Ligia* [1]. *Ligia exotica* and *L. cinerascens* are distributed in mainland China, and there are obvious differences in their physical characteristics. *L. cinerascens* is distributed in China from Dandong to Qingdao, *L. exotica* overlaps with it in some areas, and only the *L. exotica* species is distributed from Qingdao to Nantong [1].

In view of the pivotal role of *Ligia* in intertidal ecosystems, it is imperative to investigate the composition, variation, and function of their gut microbiota in relation to the functional specialization of gut segments. The gut tract is distinguished by its unique environment, which is the primary site of digestion and absorption. This environment attracts a diverse array of microbial colonization, with some commensal bacteria having the capacity to affect the physiology of the host [9]. A growing body of evidence indicates that the gut microbiota plays pivotal roles in the enhancement of egg production; the acceleration of larval development; the optimization of digestive processes; the provision of essential nutrients; the prevention of pathogen proliferation; and in the digestion, absorption, metabolism, and transformation of ingested substances within the gastrointestinal tract [10,11]. The study of microorganisms has been based on pure cultures for centuries, starting with the invention of the microscope by Antoni van Leeuwenhoek. Of the trillions of microbial species, only a small proportion, between 0.1% to 1%, can be cultured [12]. Therefore, in light of the intricate nature of these microbial organisms, the utilization of pure culture techniques to identify and ascertain their functions represents an insurmountable challenge. This can significantly limit the research and development of microbial diversity resources. The use of next-generation sequencing technology can quickly and accurately obtain a large amount of biological data and rich information for microbiological research [13]. The use of 16S rRNA gene analysis, metagenomics, metabolomics, and proteomics is important to expand our understanding of gut bacterial communities [14]. The approaches that have been developed have been used to explore the function of gut microbiota in many species, such as *Apis mellifera* [15], earthworms [16], wasps [17], and termites [18].

The aim of this research is to investigate the associations between the gut microbiota’s composition, variation, and function, as well as the functional specialization of various gut segments of *Ligia* distributed in the coast of Aoshanwei, Qingdao, Shandong Province. This will be achieved through comprehensive analyses encompassing ecological distribution, phylogeny, 16S rRNA gene sequencing, metagenomics, metabolomics, and proteomics. Meanwhile, the concentrations of common metabolites, NO_3_^−^-N and NH_4_^+^-N, and the contents of C and N were measured. Specifically, we addressed two questions: (1) How do the composition, diversity, and potential function of the gut microbiota change during the gut transit? (2) What is the functional specialization of gut segments due to the composition, diversity, and potential functions of microbiota in different gut segments?

## 2. Materials and Methods

### 2.1. Collection and Identification of L. exotica in the Aoshanwei, Qingdao, China

Live wharf roach samples were collected randomly from the coast of Aoshanwei, Qingdao, Shandong Province (120°39′50″ E, 36°20′21″ N). There was little difference in individual size. The samples were stored at −80 °C for subsequent dissection. The samples were placed on the ice, washed, and disinfected the surface with 75% alcohol and rinsed then with distilled water. The gut was divided from the middle into anterior and posterior sections, the foregut near the head and the hindgut near the tail. Foregut and hindgut samples were placed separately in sterilized centrifuge tubes, quickly frozen in liquid nitrogen, and then quickly placed for preservation at −80 °C. Three replicates were taken from the foregut and hindgut samples for subsequent omics data analysis.

The COI sequences were amplified and sequenced using COI universal primers based on published articles [1]. Gene trees were constructed based on COI sequences from 10 species from the isopods using MAFFT v7.489 sequence alignment method with default settings [19] and the ML method using the IQ-TREE v1.6.12 [20]. The K3Pu + F + I + G4 model was selected as the best, and ML analysis was performed with 1000 bootstrap replications. The resulting phylogenetic trees were visualized in FigTree v1.4.0. We used Adobe Illustrator cc v2015 to polish the phylogenetic trees.

### 2.2. 16S rRNA Gene Sequencing Analysis in the Foregut and Hindgut

Total genomic DNA was extracted by CTAB/SDS method. Concentration and purity of DNA were examined using 1% agarose gels. The DNA was diluted to 1 ng/μL and the 16S rRNA gene was amplified with a specific primer. PCR reactions were performed using transStart^®^ fastpfu DNA polymerase (TransGen Biotech, Beijing, China). Equal volumes of buffer were mixed with PCR products and detected by 2% agarose gel electrophoresis. The PCR products were mixed at equal density ratios, then purified mixed PCR products using the QIAquick@ gel extraction kit (QIAGEN, Hilden, Germany), and the sequencing library was generated using the SMRTbellTM template preprocessing kit (Pacific Biosciences, Menlo Park, CA, USA). Library quality was assessed on Qubit@ 2.0 fluorometer (Invitrogen, Carlsbad, CA, USA) and FEMTO pulse system. Finally, sequencing was performed on PacBio sequel platform.

The raw sequencing sequences were preprocessed using PacBio SMRT website, and reads were compared to reference database using the UCHIME algorithm [21] to detect chimeric sequences. Chimeric sequences were removed to obtain clean reads [22]. Finally, the microbial population composition and diversity were analyzed using Qiime2 v1.9.1 [23]. We compared the gut microbiota of *L. exotica* distributed in Qingdao, Shandong Province, in our study and South Korea based on published articles [24] to analyze the differences in the gut microbiota of the same species in different regions.

### 2.3. Metagenomic Analysis in the Foregut and Hindgut

Genomic DNA was extracted from 1 μg of sample. The genomic DNA was randomly sheared into short fragments using Covaris ultrasonic crusher, and sequencing libraries were generated. The obtained fragments were end repaired, a-tailed, and further ligated with Illumina adapter. The fragments with adapters were PCR amplified, size selected, and purified. The library was checked with Qubit v2.0 [25]. The libraries were diluted to 2 ng/uL, and the insert size of the libraries was subsequently assayed using an Agilent 2100 (Agilent Technologies, Santa Clara, CA, USA), and after meeting expectations, the effective concentration of the libraries was accurately quantified using real-time PCR. Quantified libraries were pooled and sequenced on Illumina PE150 (Illumina, San Diego, CA, USA) according to effective library concentration and data amount required.

Readfq (https://github.com/cjfields/readfq, accessed on 1 March 2024) was used for preprocessing raw data from the Illumina sequencing platform to obtain clean data for subsequent analysis. Bowtie v2 was used to send clean data to the host database to filter out reads that may come from the host source [26]. MEGAHIT v1.0 was used to perform assembly analysis on clean data and then break the assembled scaffolds from the N junctions to obtain *n*-free scaftigs [27]. MetaGeneMark v2.8 was used to perform ORF prediction for scaftigs ≥ 500 bp) of each sample and filter out the information with a length less than 100 nt in the prediction results [28]. For the ORF prediction results, CD-HIT was used to eliminate redundancy and obtain the nonredundant initial gene catalogue [29]. The clean data of each sample were compared to the initial gene catalogue using Bowtie v2, and the number of reads on which genes were compared in each sample was counted, and genes with a number of reads ≤2 were filtered out of each sample to obtain the final gene catalogue (unigenes) used for subsequent analyses.

DIAMOND v1 was used to align unigenes with those in the functional database [30,31], including Kyoto Encyclopedia of Genes and Genomes (KEGG) [32,33], Evolutionary Genealogy of Genes: Nonsupervised Orthologous Groups (eggNOG) [34], and Carbohydrate-Active enZYmes (CAZy) [35]. For the comparison results of each sequence, the comparison result with the highest score (one HSP > 60 bits) was selected for subsequent analysis [36,37]. According to the comparison results, the relative abundance of different functional levels was calculated [38]. The KEGG database was divided into 6 levels, the eggNOG database into 3 levels, and the CAZy database into 3 levels. Unigenes was aligned to the comprehensive antibiotic resistance database (CARD) (https://card.mcmaster.ca/, accessed on 10 March 2024) using the resistance gene identifier (RGI v6.0.0) [39] provided by the CARD database (RGI built-in blastp, default e-value < 1 × 10^−30^) [40]. According to the RGI alignment result and unigenes abundance information, the relative abundance of each antibiotics resistance gene (ARG) was calculated for subsequent analyses. 

### 2.4. Metabolome Analysis of the Foregut and Hindgut

Tissue samples with 200 μL of H_2_O and five ceramic beads were homogenized using the homogenizer. An amount of 800 μL methanol/acetonitrile was added to homogenized solution for metabolite extraction. The mixture was centrifuged for 20 min. The supernatant was dried in a vacuum centrifuge. For LC-MS analysis, the samples were redissolved in 100 μL acetonitrile/water solvent and centrifuged at 14,000× *g* at 4 °C for 15 min; then, the supernatant was injected.

LC-MS/MS analysis was performed using an UHPLC coupled to a quadrupole time of flight. The raw MS data were converted to MzXML files using ProteoWizard [41]. XCMS software (https://xcmsonline.scripps.edu, accessed on 14 March 2024) was used for peak alignment, retention time correction, and peak area extraction [42]. The data extracted by XCMS were firstly subjected to metabolite structure identification and data pre-processing, followed by experimental data quality evaluation and finally data statistical analysis.

After sum-normalization, the processed data were analyzed by R package (ropls), where they were subjected to multivariate data analysis, including PCA and OPLS-DA analysis. The VIP value of each variable in the OPLS-DA model was calculated to indicate its contribution to the classification. The T test was applied to determine the significance of differences between two groups. VIP > 1 and *p* value < 0.05 were used as thresholds to screen for significantly different metabolites. Pearson’s correlation analysis was performed to determine the correlation between two variables.

### 2.5. Proteomic Analysis of the Foregut and Hindgut

Samples were first homogenized using the MP FastPrep-24 homogenizer, and then SDT buffer was added. Samples were immediately diluted in 6 M guanidine-10 mM dithiothreitol and mixed at 600 rpm for 1.5 h. After the samples cooled to room temperature, IAA was added with the final concentration of 20 mM into the mixture, and the samples were incubated for 30 min in darkness. Next, the samples were filtered using the filters. Finally, trypsin was added to the sample and incubated at 37 °C for 15–18 h, and the resulting peptides were collected as a filtrate. The peptides of each sample were desalted on C18 cartridges, concentrated by vacuum centrifugation, and reconstituted in 40 µL of 0.1% formic acid. The peptides from each sample were analyzed by mass spectrometer connected to Vanquish Neo System liquid chromatography in the data independent acquisition (DIA) mode. DIA data were analyzed with DIA-NN v1.8.1. All reported data were based on 99% confidence for protein identification as determined by false discovery rate (FDR) ≤ 1%.

The data were used to performing hierarchical clustering analysis using the Cluster 3.0 (http://dnagarden.hgc.jp/en/doku.php/software, accessed on 20 March 2024) and Java TreeView software (http://jtreeview.sourceforge.net, accessed on 26 March 2024). Protein sequences were searched using the InterProScan v5.50-84.0 to identify protein domain signatures from the InterPro member database Pfam. The protein sequences of the selected differentially expressed proteins were locally searched using the NCBI BLAST^+^ v2.2.28 and InterProScan v5.50-84.0 to find homologue sequences. Gene ontology (GO) terms were mapped and sequences were annotated using the software program Blast2GO v6.0. The studied proteins were blasted against the online KEGG database to retrieve their KEGG orthology identifications.

### 2.6. Experimental Determination of Physico-Chemical Properties

To determine the content of common metabolites of the foregut and hindgut samples, after slowly thawing the samples at 4 °C, an appropriate amount of the samples was added to the pre-cooled methanol/acetonitrile/water solution and then vortexed to mix the samples, which were then sonicated at low temperature, allowed to stand and centrifuged, and then the supernatants were collected and vacuum dried. The samples were separated by Agilent 1290 Infinity LC (Agilent Technologies, Santa Clara, CA, USA) ultra-high performance liquid chromatography HILIC and C18 columns. The samples were placed in a 4 °C automatic injector during the entire analysis process. To avoid the influence of fluctuating instrumental detection signals, the samples were analyzed sequentially in random order. Acetonitrile solution was added to redissolve during mass spectrometry. After centrifugation at 4 °C for 15 min at 14,000× *g*, the supernatant was taken for analysis. Mass spectrometry was performed by AB 6500+ QTRAP mass spectrometer (SCIEX, Framingham, MA, USA).

To ascertain the NO_3_^−^-N and NH_4_^+^-N content in the foregut and hindgut samples, visible spectrophotometry was employed in accordance with the instructions provided by the manufacturer. A sample of 0.1 g was prepared, with three replicates for both the foregut and hindgut samples. The absorbance values of the foregut and hindgut samples were measured using the detection kit (Suzhou Grace Biotechnology Co., Ltd., Suzhou, China) and a spectrophotometer. The NO_3_^−^-N and NH_4_^+^-N content in the foregut and hindgut samples were then calculated using the formulas NO_3_^−^-N (μg/g) = 148.9 × (ΔA + 0.0005) ÷ W and NH_4_^+^-N (μmol/g) = 2.1 × (ΔA–0.0154) ÷ W, respectively.

To ascertain the content of C and N in the foregut and hindgut samples, we ground the dried samples and tested the samples using the Vario ELIII fully automatic elemental analyzer to achieve full combustion. The sample underwent high-temperature combustion (oxidized at 1150 °C and reduced at 850 °C), followed by gas separation using adsorption–desorption columns with specific temperature parameters: CO_2_ column (40 °C adsorption/90 °C release), H_2_O column (40 °C adsorption/150 °C release), and SO_2_ column (140 °C adsorption/240 °C release). Elemental content was then quantified using a TCD detector.

## 3. Results

### 3.1. Collection and Identification of L. exotica

**Figure 1:**
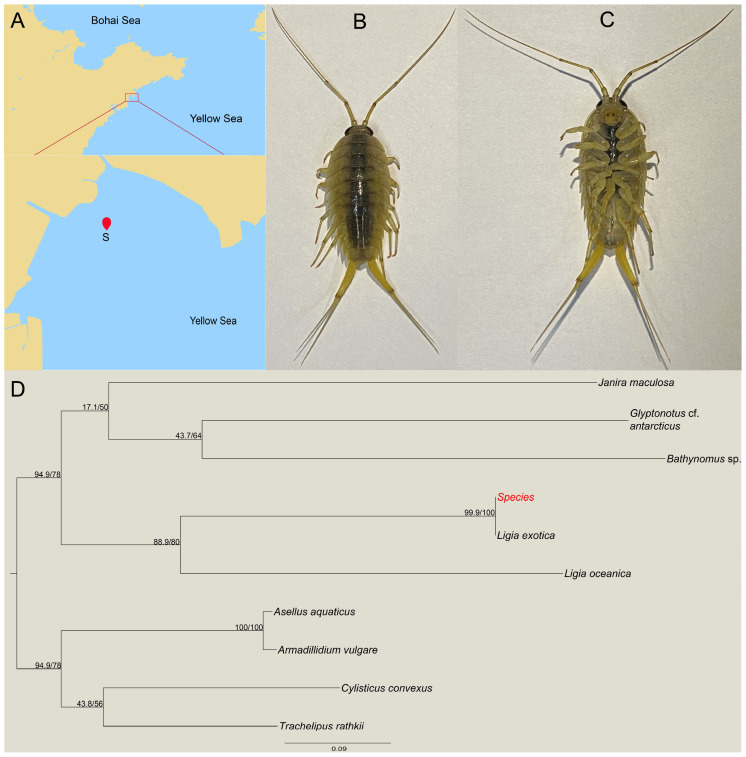
Collection and identification of *Ligia exotica* in the coast of Aoshanwei, Qingdao, Shandong province. The red dot represents sample collection site (**A**); the morphology of *L. exotica* dorsal (**B**); and abdomen (**C**). Phylogenetic tree of isopoda based on the COI sequences; the “*Species*” represent the species we studied (**D**).

Samples were collected from the coast of Aoshanwei, Qingdao City, Shandong Province (120°39′50″ E, 36°20′21″ N) (Figure 1A). The specimen of the wharf roach displays a dark gray body with a long second antenna, whip segments of 30 or more, a caudal limb, and internal and external appendages that are elongated (Figure 1B,C). In order to facilitate further species identification, the COI sequences were amplified and sequenced. Subsequently, a phylogenetic tree of isopods was constructed based on the COI sequences (Figure 1D). The wharf roach identified at the coast of Aoshanwei, Qingdao, was confirmed to be *L. exotica*, which aligns with the known ecological distribution of this species.

### 3.2. The Composition of Gut Microbial Community

**Figure 2:**
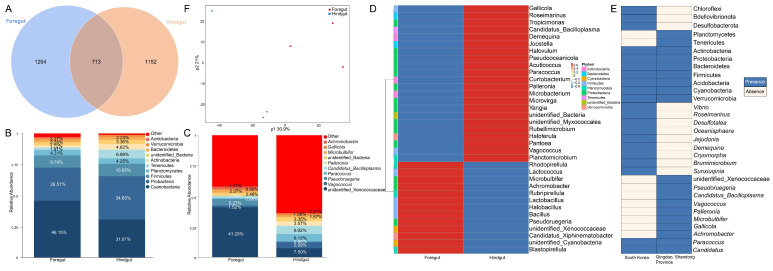
The 16S rRNA gene sequencing analysis in the foregut and hindgut of *L. exotica*. Venn diagram of gut bacteria in the foregut and hindgut samples of *L. exotica* (**A**). Histogram of relative abundance of species at the phylum (**B**) and genus (**C**) level in the foregut and hindgut of *L. exotica*. The abundance clustering heatmap of bacteria at the genus level in the foregut and hindgut of *L. exotica* (**D**). The presence and absence of gut microbiota between *L. exotica* in South Korea and *L. exotica* in Qingdao, Shandong Province. Absence or presence of a certain gut microbes is marked in yellow or blue (**E**). The β diversity of gut microbiota in the foregut and hindgut of *L. exotica* based on the PLS-DA method (**F**).

In total, 37 phyla, 44 classes, 103 orders, 197 families, 479 genera, and 355 species were obtained from the sequencing of the gut microbes of *L. exotica*. In total, 713 operational taxonomic units were identified as being shared between the foregut and hindgut samples. Of these, 1264 were specific to foregut samples and 1152 were specific to hindgut samples (Figure 2A).

Cyanobacteria (38.76%), Proteobacteria (30.58%), Firmicutes (9.90%), Tenericutes (4.45%), Planctomycetes (4.28%), Actinobacteria (3.17%), unidentified_Bacteria (2.97%), Bacteroidetes (2.25%), Verrucomicrobia (1.35%), and Acidobacteria (0.42%) were the most abundant ten phyla detected in the gut bacterial communities of *L. exotica* (Figure 2B). In these gut bacterial communities, the percentage distributions of major phyla in the foregut were as follows: Cyanobacteria (46.15%), Proteobacteria (26.51%), Firmicutes (9.74%), Tenericutes (1.91%), and Actinobacteria (1.72%). The percentage distributions of major phyla in the hindgut were as follows: Proteobacteria (34.65%), Cyanobacteria (31.07%), Firmicutes (10.05%), Tenericutes (6.99%), and Actinobacteria (4.62%) (Table 1). Unidentified_Xenococcaceae (24.35%), *Candidatus_Bacilloplasma* (4.31%), *Paracoccus* (3.50%), *Vagococcus* (3.31%), *Pseudoruegeria* (2.94%), unidentified_Bacteria (2.91%), *Palleronia* (1.81%), *Microbulbifer* (1.97%), *Gallicola* (1.23%), and *Achromobacter* (0.99%) were the most abundant ten families, genera, and species detected in the gut bacterial communities of *L. exotica* (Figure 2C,D). In these gut bacterial communities, the percentages of major families and genera in the foregut were as follows: unidentified_Xenococcaceae (41.20%) and *Pseudoruegeria* (5.27%). The percentages of major families, genera, and species in the hindgut were as follows: unidentified_Xenococcaceae (7.50%), *Candidatus_Bacilloplasma* (6.92%), *Paracoccus* (6.12%), and *Vagococcus* (5.09%) (Table 2).

A comparison of the gut microbiota of *L. exotica* distributed in South Korea and Qingdao, Shandong Province, revealed significant differences in the composition of the gut microbiota between the species from the two locations (Figure 2E). Chloroflexi, Bdellovibrionota, and Desulfobacterota were the unique phyla detected in the gut bacterial communities of *L. exotica* distributed in South Korea. Planctomycetes and Tenericutes were the unique phyla detected in the gut bacterial communities of *L. exotica* distributed in Qingdao, Shandong Province. The gut microbiota of this species in the two regions differed considerably at the family, genus and species levels. They only shared two genera: *Paracoccus* and *Candidatus*.

### 3.3. The Diversity of Gut Microbial Community

The α diversity indices of the gut microbiota of *L. exotica* are shown in Table 3. The Shannon and Simpson indices of the foregut and hindgut samples were not significantly different, and the foregut index value was slightly greater than the hindgut index value. The ACE and Chao 1 indices were found to be lower in the foregut than in the hindgut. Based on the PLS-DA method, the β diversity of the gut microbiota was analyzed (Figure 2F), and it was found that the foregut and the hindgut bacteria clustered separately, indicating that there were significant differences in the community structure of the foregut and the hindgut bacteria.

### 3.4. Metagenomic Analysis of Foregut and Hindgut

**Figure 3:**
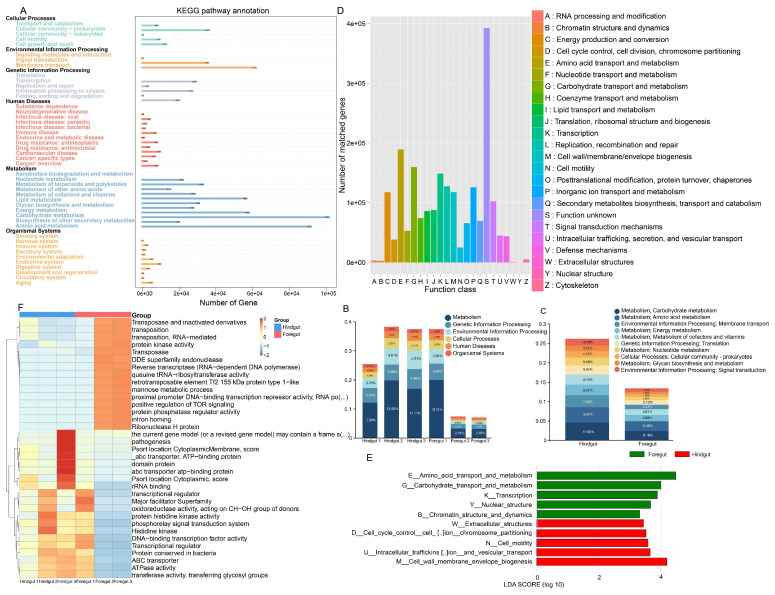
KEGG and EggNOG analysis in metagenomic sequencing of the foregut and hindgut of *L. exotica*. Number of genes in metabolic pathways annotated to KEGG level 2 by the gut microbiota of *L. exotica* (**A**). Relative abundance on the first (**B**) and second (**C**) levels of KEGG in the foregut and hindgut of *L. exotica*. The functional proteins contained in the *L. exotica* gut microbiota (**D**). Distribution of functional LDAs based on EggNOG level 1 differences in the foregut and hindgut of *L. exotica* (**E**). Heatmap of horizontal distribution based on EggNOG level 2 (**F**).

The KEGG annotation of the gut microbiota from the foregut and hindgut of *L. exotica* revealed 46 significantly different pathways (Figure 3A). The pathway with the highest number of annotated genes was metabolism (foregut: 5.77%; hindgut: 10.70%), followed by genetic information processing (foregut: 1.92%; hindgut: 3.79%), environmental information processing (foregut: 1.50%; hindgut: 3.36%), cellular processes, human diseases, and the lowest number of genes at organismal systems (Figure 3B). Carbohydrate metabolism (foregut: 6.18%; hindgut: 11.80%), amino acid metabolism (foregut: 6.35%; hindgut: 9.94%), membrane transport (foregut: 3.32%; hindgut: 7.86%), energy metabolism (foregut: 3.88%; hindgut: 6.87%), metabolism of cofactors and vitamins (foregut: 3.61%; hindgut: 6.70%), translation (foregut: 3.12%; hindgut: 5.84%), nucleotide metabolism (foregut: 2.08%; hindgut: 5.08%), cellular community prokaryotes (foregut: 1.83%; hindgut: 4.10%), glycan biosynthesis and metabolism (foregut: 1.54%; hindgut: 3.72%), and signal transduction (foregut: 1.97%; hindgut: 4.20%) were the predominant signaling pathways (Figure 3C). The proportion of gut microbiota engaged in macromolecular synthesis and catabolism, as well as fermentation, is presented in Tables S1 and S2.

Based on the EggNOG analysis (Figure 3D), the highest number of annotated genes was found in amino acid transport and metabolism (foregut: 5.1%; hindgut: 4.6%), followed by carbohydrate transport and metabolism (foregut: 3.5%; hindgut: 4.1%); transcription (foregut: 3.3%; hindgut: 3.9%); replication, recombination, and repair (foregut: 2.6%; hindgut: 4.2%); and translation, ribosomal structure, and biogenesis (foregut: 1.9%; hindgut: 3.7%). Foregut genes were significantly enriched in amino acid transport and metabolism, carbohydrate transport and metabolism, transcription, nuclear structure, and chromatin structure and dynamics. Hindgut genes were significantly enriched in extracellular structures, cell cycle control, cell division and chromosome partitioning, cell motility, intracellular transport, secretion and vesicular translocation, and cell wall membrane/envelope biogenesis (Figure 3E).

At level 2, the top ten relative pathways in the foregut includeded ribonuclease H protein, intron homing, reverse transcriptase (RNA-dependent DNA polymerase), proximal promoter DNA-binding transcription repressor activity, the mannose metabolic process, positive regulation of TOR signaling, DDE superfamily endonuclease, transposition, transposase and inactivated derivatives, transposition, and RNA-mediated. The top ten relative pathways in the hindgut included the transcriptional regulator, ATPase activity, ABC transporter, rRNA binding, the phosphorelay signal transduction system, transferase activity, transferring glycosyl groups, DNA-binding transcription factor activity, protein conserved in bacteria, transcriptional regulator, and histidine kinase (Figure 3F and Table S3).

**Figure 4:**
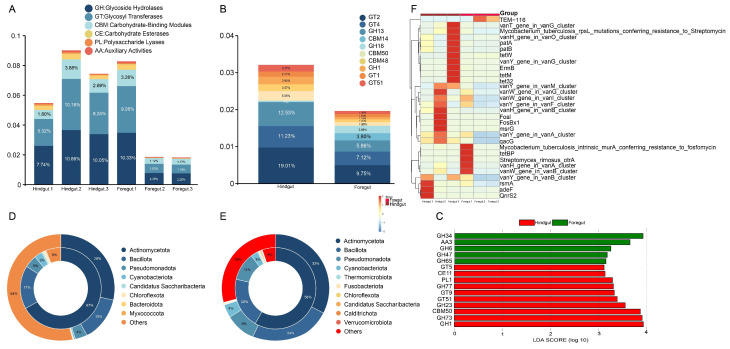
CAZy and CARD analysis in metagenomic sequencing of the foregut and hindgut of *L. exotica*. Relative abundance of the six functions of CAZy in the foregut and hindgut of *L. exotica* (**A**) and relative abundance of the level 2 of CAZy in the foregut and hindgut of *L. exotica* (**B**). Differential functional LDA distribution based on CAZy level 1 in the foregut and hindgut of *L. exotica* (**C**). Attribution analysis of resistance genes in the foregut (**D**) and hindgut (**E**) at the phylum. Heatmap of top 30 antibiotic resistance gene abundance clustering in the foregut and hindgut of *L. exotica* (**F**).

A total of 234 different results were identified based on the CAZy functional annotation. As shown in Figure 4A, among the six functions, glycoside hydrolases (GHs) had the highest relative abundance, followed by glycosyl transferases (GTs), carbohydrate-binding modules (CBMs), carbohydrate esterases (CEs), polysaccharide lyases (PLs), and auxiliary activities (AAs). At the family level, the top ten functional enzymes in relative abundance were GT2 (foregut: 9.75%; hindgut: 19.01%), GT4 (foregut: 7.12%; hindgut: 11.23%), GH13 (foregut: 5.86%; hindgut: 12.53%), CBM14 (foregut: 3.80%; hindgut: 0.16%), GH18 (foregut: 3.86%; hindgut: 0.60%), CBM50 (foregut: 1.90%; hindgut: 5.05%), CBM48 (foregut: 1.45%; hindgut: 3.57%), GH1 (foregut: 1.19%; hindgut: 3.95%), GT51 (foregut: 1.36%; hindgut: 3.27%), and GT1 (foregut: 1.64%; hindgut: 2.71%) (Figure 4B). The highest relative abundance in the GH family was GH13, GH18, and GH1. The highest relative abundance in the GT family was GT2, GT4, GT1, and GT51. The highest relative abundance in the CBM family was CBM14, CBM50, and CBM48. The main functional enzymes with higher relative abundance in the foregut than in the hindgut were GH18 and CBM14, and the main functional enzymes with higher relative abundance in the hindgut than in the foregut were GT2, GH13, and GT4 (Figure 4B). The results of the LEfSe analysis indicated that the GH gene family exhibited significant differences between the foregut and hindgut (LDA > 3, *p* < 0.05). The GH34, AA3, GH6, GH47, and GH65 genes were found to be being significantly enriched in the foregut, while the GT5, CE11, PL1, GH77, GT9, GT51, GH23, CBM50, GH73, and GH1 genes were found to be significantly enriched in the hindgut (Figure 4C).

In the foregut microbiota, Actinomycetota had the highest percentage of 28% and carried 67% of the ARGs, Bacillota had 13% and carried 17% of the ARGs, Pseudomonadota had 4% and carried 5% of the ARGs, and Cyanobacteriota had 0.5% and carried 3% of the ARGs (Figure 4D). In the hindgut microbiota, Actinomycetota had the highest percentage of 33% and carried 58% of the ARGs, Bacillota had 24% and carried 20% of the ARGs, Pseudomonadota had 9% and carried 11% of the ARGs, and Cyanobacteriota had 4% and carried 3% of the ARGs (Figure 4E). In the foregut and hindgut, the most abundant ARG was the vancomycin class, followed by tetracyclines, macrolides-lincosamide-streptogramin, chemical disinfectants, rsmA, and lincosamides (Figure 4F). However, the abundance of ARGs was higher in the hindgut than in the foregut (Table S4).

### 3.5. Metabolome Analysis of Foregut and Hindgut

**Figure 5:**
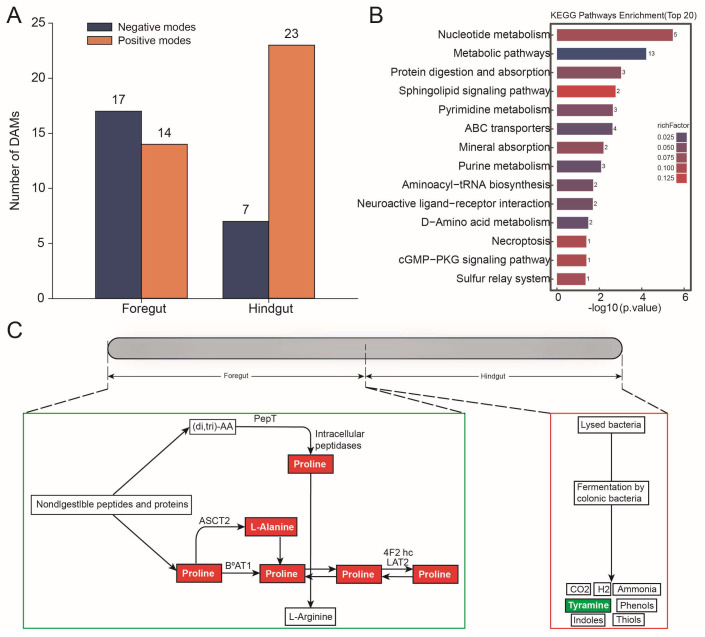
Metabolome analysis in the foregut and hindgut of *L. exotica*. Amounts of different metabolites in the foregut and hindgut of *L. exotica* (**A**). KEGG enrichment analysis of differential metabolites (**B**). Protein digestion and absorption pathway involved in differential metabolites in the foregut and hindgut of *L. exotica*, where red represents metabolites that represent significant upregulation of abundance in the foregut, while green represents metabolites that represent significant upregulation of abundance in the hindgut (**C**).

In the foregut, 31 differential metabolites were significantly upregulated in abundance; in the hindgut, there were 30 differential metabolites that were significantly upregulated in abundance (OPLS-DA VIP > 1, *p* value < 0.05, and Fold change > 1 or Fold change < 1) (Figure 5A and Tables S5 and S6). The differential metabolites were found to be significantly enriched in the protein digestion and absorption pathway (Figure 5B). In this pathway, the abundance of alanine (Fold change = 0.696; *p*-value = 0.021; VIP = 2.437) and the abundance of proline (Fold change = 0.429; *p*-value = 0.038; VIP = 10.390) were significantly upregulated in the foregut based on the negative modes, and the abundance of tyramine (Fold change = 1.724; *p*-value = 0.013; VIP = 4.808) was significantly upregulated in the hindgut based on the positive modes (Figure 5C).

### 3.6. Proteome Analysis of Foregut and Hindgut

**Figure 6:**
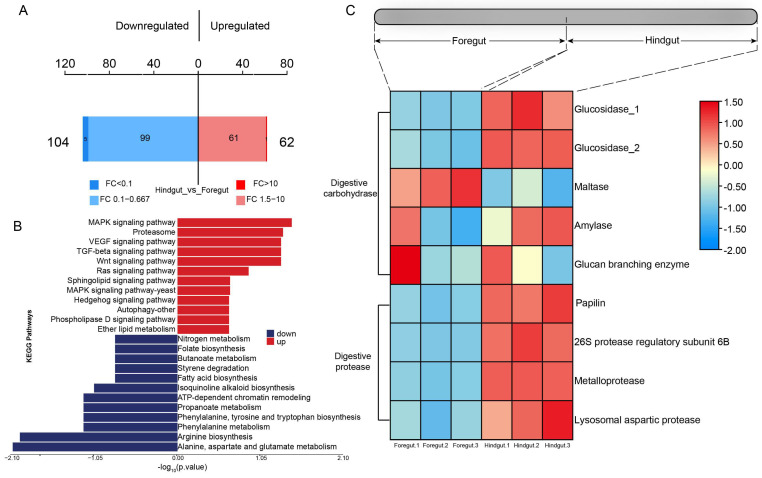
Proteome analysis in the foregut and hindgut of *L. exotica*. The bar chart of protein quantitative differences (**A**). Red color refers to the number of proteins significantly upregulated in the hindgut, and blue color refers to the number of proteins significantly upregulated in the foregut. KEGG diagram of significantly upregulated and downregulated differentially expressed proteins (**B**). Heatmap illustrating digestive enzyme expression among different gut segments, where red boxes indicate high expression patterns of proteins in different gut segments, and blue boxes indicate low expression patterns of proteins in different gut segments (**C**).

Based on further proteome analysis for different gut segments, it was found that a total of 166 differential proteins were identified, with 104 significantly upregulated in the foregut and 62 significantly upregulated in the hindgut (*p*-value < 0.05) (Figure 6A). Proteins associated with the “PI3K-Akt signaling pathway”, “Focal adhesion”, the “MAPK signaling pathway”, “Proteasome”, the “AMPK signaling pathway”, the “Wnt signaling pathway”, the “TGF-beta signaling pathway”, and the “VEGF signaling pathway” were significantly upregulated in the hindgut. Proteins associated with “Alanine, aspartate and glutamate metabolism” and “Arginine biosynthesis” were significantly upregulated in the foregut (Figure 6B). The highest activities of glucosidase, amylase, and protease were exhibited in the hindgut, with amylase also showing relatively high activity in the foregut, and maltase activities were relatively elevated in the foregut (Figure 6C).

### 3.7. Analysis of Changes in Physico-Chemical Properties

**Figure 7:**
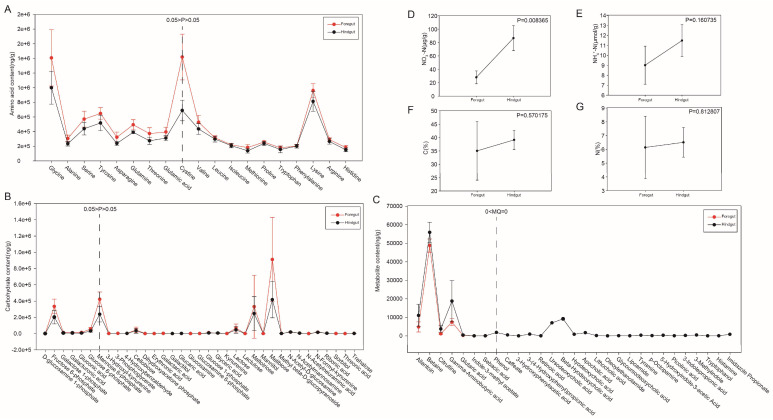
The determination of physico-chemical properties in the foregut and hindgut. The content of amino acids commonly found in the foregut and hindgut (**A**). The content of carbohydrate commonly found in the foregut and hindgut (**B**). The content of metabolites significantly more abundant in the hindgut than in the foregut and the content of metabolites present only in the hindgut (**C**). The concentrations of NO_3_^−^-N and NH_4_^+^-N and the elemental percentages of C and N in the foregut and hindgut (**D**–**G**).

The contents of 19 amino acids in the foregut and hindgut are shown in Figure 7A and Table S7. The former exhibited a markedly higher concentration of nine amino acids (*p*-value < 0.05), including Glycine (Gly), Alanine (Ala), Serine (Ser), Tyrosine (Tyr), Asparagine (Asn), Glutamine (Gln), Threonine (Thr), Glutamic acid (Glu), and Cystine (Cys). While the remaining 10 amino acids did not exhibit a statistically significant difference between the foregut and hindgut, they were all present in greater quantities within the foregut than within the hindgut. The content of carbohydrates commonly found in the foregut and hindgut was measured (Figure 7B and Table S8). Among them, a number of common intermediates of carbohydrate metabolism were found to be significantly more abundant in the foregut than in the hindgut (*p*-value < 0.05), including D-glucosamine 1-phosphate, fructose 6-phosphate, galactose 1-phosphate, galacturonic acid, gluconic acid, glucose 6-phosphate, and mannose 6-phosphate. Furthermore, our findings revealed that certain metabolites exhibited significantly higher abundance in the hindgut compared to the foregut, while others were exclusively present in the hindgut (Figure 7C and Table S9). The contents of allantoin, betaine, citrulline, gamma-aminobutyric acid, glutaric acid, indole-3-methyl acetate, and sebacic acid were significantly higher in the hindgut than in the foregut (*p*-value < 0.05). Furthermore, regarding phenol, caffeate, 3-hydroxyphenylacetic acid, 3-(4-hydroxyphenyl)propionic acid, retinoic acid, ursodeoxycholic acid, beta-hyodeoxycholic acid, hyodeoxycholic acid, apocholic acid, lithocholic acid, oleoylethanolamide, glycoursodeoxycholic acid, lipoamide, tyramine, p-octopamine, 5-hydroxyindole-3-acetic acid, picolinic acid, 3-indolepropionic acid, 3-methylindole, tryptophanol, histamine, and imidazole propionate, these 22 metabolites could only be found in the hindgut.

The concentrations of NO_3_^−^-N and NH_4_^+^-N, along with the elemental percentage of C and N in the foregut and hindgut, are illustrated in Figure 7D–G and Table S10, respectively. The concentrations of NO_3_^−^-N were significantly higher in the hindgut than in the foregut (*p*-value < 0.05). While the concentrations of NH_4_^+^-N, along with the elemental percentages of C and N, did not exhibit statistically significant differences between the foregut and hindgut, they were all present in greater quantities within the hindgut than within the foregut.

## 4. Discussion

The observed variations in microbial communities between the three replicate samples of the foregut and the hindgut may be attributable to the fact that the *L. exotica* were not fasted prior to dissection, thereby resulting in individual differences. It is noteworthy that the same sample treatment method was employed in related studies [43]. The *L. exotica* gut tract harbors a diverse bacterial community primarily composed of Cyanobacteria, Proteobacteria, Firmicutes, and Tenericutes. These microorganisms have been shown to greatly influence the species’ survival, behavior, spawning, longevity, and larval development [44]. They can enhance the survival and adaptability of *L. exotica* in dynamic intertidal environments. Cyanobacteria experienced a reduction in abundance upon traversing the gut tract, implying their potential role as a food source that is subject to digestion. *L. exotica* prefers to feed on algae [6], and algae present in their living environment are ingested and retained in their guts [45], which are amplified by universal primers for the 16S rRNA gene to form a large number of Cyanobacteria. The eating dynamics of *L. exotica* and living environment also create its strong adaptability to its environment. The abundance of Planctomycetes and Bacteroidetes remained relatively constant throughout the process of gut transit. Planctomycetes are a group of bacteria known for their ability to convert nitrate nitrogen to ammonia nitrogen in anoxic environments [46], which is a property that may allow *L. exotica* to play vital roles in mitigating seawater acidification, maintaining the health of marine ecosystems, protecting biodiversity, safeguarding fisheries resources, and reducing negative impacts on human society [47]. Bacteroidetes are able to break down complex carbohydrates, including cellulose, pectin, and glycoproteins [48]. The high abundance of Proteobacteria, Firmicutes, and Actinobacteria was found in the hindgut. Proteobacteria dominate the gut microbiota of invertebrates; this may be attributed to the wide distribution of these bacteria [49]. It has been shown that N enriched in gut mucus promotes rapid growth of Proteobacteria [50]. The high percentage of N in the hindgut explains why the abundance of Proteobacteria in the hindgut was higher than in the foregut. Enzymes produced by Proteobacteria are instrumental in the digestion of starch and hemicellulose. Furthermore, Proteobacteria are capable of providing essential nutrients such as amino acids and nitrogen [51]. The dominance of Proteobacteria could improve the ability of the hindgut to digest and assimilate food [52]. Firmicutes are mainly associated with the metabolism of gut substances by encoding enzymes such as 2-oxoacid ferredoxin oxidoreductase and triosephosphate isomerase, which are involved in the catabolism of carbohydrates and lipids [53]. A markedly elevated ratio of Firmicutes to Bacteroidetes was observed in the hindgut relative to the foregut. This can lead to the enrichment of gut bacterial communities with high glycolytic activity, thereby enhancing the capacity to acquire energy and supply nutrients [54]. Actinobacteria are a large and diverse group of bacteria that produce a wide range of secondary metabolites, many of which have important biological activities, including antibiotics [55,56]. This also resulted in the higher abundance of ARGs in the hindgut than in the foregut. This affects the diversity and abundance of bacterial communities in different gut segments. Xenococcaceae are classified within the Cyanophyta, Cyanophyceae, and are thus designated as cyanobacteria [57]. Xenococcaceae experienced a reduction in abundance upon traversing the gut tract. This finding is in accordance with the results of the comparative analysis of the foregut and hindgut of Cyanobacteria, which were previously discussed. The abundance of *Candidatus_Bacilloplasma* was found to be higher in the hindgut. It plays a significant role in facilitating the digestion of food through the breakdown of fiber [58]. The geographical location of an organism has been demonstrated to exert a significant influence on the composition of its gut microbiota [59]. This is determined by the different living environments in different geographic regions. Geography is a major determinant of gut microbial composition.

The bacterial functional groups were significantly altered during the gut transit. KEGG and EggNOG analysis confirmed that metabolism is a principal function of the gut microbes, particularly carbohydrate metabolism and amino acid metabolism. Furthermore, gut microbes associated with the synthesis and catabolism of macromolecules, including starch, cellulose, chitin, and glycogen, were also distributed in both the foregut and hindgut. However, fermentation functional groups were higher in the hindgut than foregut. Hindgut bacteria possess genes associated with cell motility, intracellular transport, secretion, and vesicular translocation, and they exhibit abundant ATPase activity, ABC transporter activity, and transferase activity, which are associated with the excretion of digested organic molecules from the hindgut. Related studies have corroborated the notion that proteins with transferase activity can mediate the exocytosis of organic molecules [60]. The high abundance of cell wall_membrane_envelope biogenesis was higher in the hindgut and may be related to transmembrane transport and the efflux of antibiotics [61]. This finding is in accordance with the aforementioned results, which indicated that the elevated abundance of Actinobacteria in the hindgut resulted in the accumulation of antibiotics.

Gut microbiota has an extensive and diverse CAZy library that assembles or breaks down oligosaccharides and polysaccharides [62]. The GHs are a class of enzymes involved in many biological processes by hydrolyzing glycosidic bonds in glycosides and play an important role in the degradation of carbohydrates and the assembly of glycoproteins [63]. The CBMs are a class of protein structural domains attached to GH enzymes that do not have catalytic activity but function as substrate-binding modules; they enhance the catalytic function of the enzyme by targeting the enzyme to the substrate and increasing the contact between the substrate and the enzyme [64]. The relative abundances of GH18 and CBM14 were found to be higher in the foregut. GH18 is a chitinase that hydrolyses beta-1,4-linkages in chitin. It is widely expressed in prokaryotes and eukaryotes and is involved in biological processes such as cell wall degradation and remodeling, parasitic organism invasion, and plant defense responses. Certain bacteria and plants that do not produce chitin use chitinases to convert insoluble chitin into a metabolizable nutrient source and to defend against chitin-bearing pathogens, respectively [65]. CBM14 typically binds to enzymes or other proteins, facilitating their localization to specific carbohydrate substrates [66]. The combination of GH18 and CBM14 in the foregut is important for the conversion of insoluble chitin from ingested material to metabolizable nutrient sources. The relative abundances of GT2, GH13, and GT4 were found to be higher in the hindgut. GT2 and GT4 are instrumental in the breakdown and synthesis of disaccharides, oligosaccharides, and polysaccharides; glycosyl transfer and modification; cell signaling; and cell surface glycosyl recognition [67]. GH13 contains a variety of specific enzymes whose primary function is to catalyze the breakdown of polysaccharides (e.g., starch and glycogen) into monosaccharides (e.g., glucose). These enzymes play a significant role in carbohydrate and energy metabolism [68].

We identified and analyzed differential metabolites and proteins between foregut and hindgut samples and experimentally determined the physico-chemical properties of different gut segments. Differential metabolites were simultaneously significantly enriched in protein digestion and the absorption pathway. In this pathway, several amino acid abundances were significantly upregulated in the foregut (*p*-value < 0.05). Differential proteins were also significantly enriched in amino acid metabolism in the foregut, which corresponds to the function of the foregut to facilitate the breakdown of ingested macromolecules such as proteins and peptides into smaller molecules such as amino acids [69,70]. The abundance of tyramine was significantly increased in the hindgut, which is a biogenic trace amine that is generated via the decarboxylation of tyrosine [71]. Further catabolism of tyrosine produces tyramine, phenylpropanol, and p-coumarin, and the production of these substances is important for the normal life of the individual [72]. Differential proteins in the hindgut were significantly enriched in a variety of signaling pathways, suggesting that the hindgut plays more complex functions. Different gut segments provide partial enzyme activity for food digestion and absorption, while the remaining portion may be contributed by symbiotic microorganisms [47].

**Figure 8:**
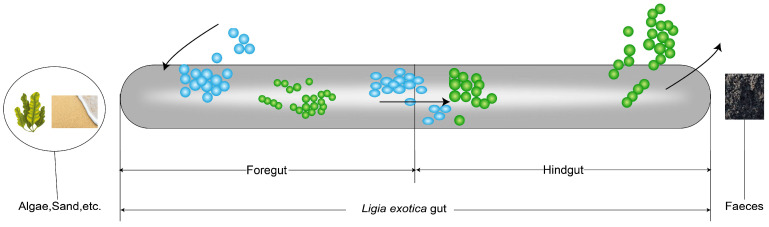
The diagram of the process of ingested food digestion by *L. exotica*.

Protein is one of the essential nutrients for animals. Dietary protein is digested in the gastrointestinal tract and broken down into amino acids before it can be further absorbed and utilized [73]. The results of the experiment verified that the amino acid concentrations were higher in the foregut, implying that the amino acids metabolism process is more active in foregut than in hindgut. Some amino acid catabolites were present only in the hindgut, such as citrulline, and these gut microbes are able to synthesize citrulline from nitrogenous substances ingested from food, such as glutamic acid, aspartic acid, and proline [74]. The metabolites 5-hydroxyindole-3-acetic acid, picolinic acid, 3-indolepropionic acid, 3-methylindole, and tryptophanol are catabolites of tryptophan [75]. Histamine and imidazole propionate are catabolites of histidine [76]. The concentrations of the intermediates of carbohydrate metabolism were markedly elevated in the foregut, implying that carbohydrate metabolic processes are more active in the foregut than in the hindgut. Among these carbohydrates, D-Glucosamine-1-phosphate is involved in glucosamine-related metabolic processes and is one of the precursors for glucosamine synthesis. It is also involved in a number of glucose metabolic pathways, including gluconeogenesis and glycolysis [77]. Fructose-6-phosphate is an important intermediate in glycolysis and gluconeogenesis pathways [78]. Galactose-1-phosphate is an intermediate metabolite of galactose metabolism [79]. Galacturonic acid is a monosaccharide, which is the constituent unit of pectic acid. There are two main metabolic pathways of galacturonic acid in prokaryotes: the oxidation pathway [80] and the isomerization pathway [81]. Gluconic acid is an aldonic acid derived from β-D-glucose via a site-specific oxidation [82]. Glucose 6-phosphate is involved in biochemical pathways such as the pentose phosphate pathway and glycolysis. It is catalyzed by glucose phosphate isomerase in the glycolysis pathway to form fructose-6-phosphate for the next step [83]. Mannose is a monosaccharide and a six-carbon sugar. Mannose is phosphorylated by hexokinase to become mannose-6-phosphate. Mannose-6-phosphate is capable of disrupting the normal glucose metabolic process, thus affecting the glucose metabolic process [84]. The experiment results showed that the metabolite contents related to the fermentation, absorption, and assimilation of substances by gut microbiota were higher in the hindgut than in the foregut or only in the hindgut. The undigested amino acids are often fermented into many bacterial metabolites or end products, including short-chain fatty acids, hydrogen sulfate, and ammonia [85]. Among them, the most important metabolites after bacterial fermentation are short-chain fatty acids, including acetic acid, propionic acid, butyric acid, valeric acid, isovaleric acid, and isobutyric acid [86]. Among them, acetic acid, propionic acid, and butyric acid are metabolites of the fermentation of carbohydrates by gut bacteria [87]. In our study, the contents of short-chain fatty acids such as gamma-aminobutyric acid and glutaric acid were significantly higher in the hindgut than in the foregut (*p*-value < 0.05). In addition, metabolites such as phenol [88], caffeate [89], 3-hydroxyphenylacetic acid [90], 3-(4-Hydroxyphenyl)propionic acid [91], retinoic acid [92], tyramine [93], and p-octopamine [94] associated with fermentation were present only in the hindgut. These experimental data show that the gut microorganisms in the hindgut facilitate the breakdown and transformation of nutrients in food through fermentation, resulting in the production of short-chain fatty acids, vitamins, and other beneficial substances that are subsequently absorbed and utilized by the individual. The metabolites of oleoylethanolamide, glycoursodeoxycholic acid, and lipoamide are involved in lipid metabolism [95,96]. The metabolites of ursodeoxycholic acid, beta-hyodeoxycholic acid, hyodeoxycholic acid, apocholic acid, and lithocholic acid are involved in bile acid metabolism [97,98,99]. Bile acids are essential for the digestion and absorption of fats. They emulsify fats, facilitating their dispersion in the small intestine and enabling more effective breakdown and absorption by digestive enzymes [100]. When food with a high nitrogen content is ingested into the gut transport process, gut microbes break down these proteins to produce NH_3_, which is further oxidized to form NO_2_^−^ and NO_3_^−^. This explains why the content of NO_3_^−^-N was significantly higher in the hindgut than in the foregut. The incomplete digestion of carbohydrates and proteins in the foregut leads to some undigested carbohydrates, amino acids, and peptides reaching the hindgut. This leads to a higher percentage of C and N in the hindgut compared to the foregut. Furthermore, proteins and amino acids that are not digested by the foregut are subjected to microbial fermentation in the hindgut, resulting in the production of NH_4_^+^-N. This leads to a higher concentration of NH_4_^+^-N in the hindgut than in the foregut. Utilizing a comprehensive data analysis and experimental results, a food digestion and absorption model of *L. exotica* gut was constructed (Figure 8). That is, the foregut appears to be the primary site of food digestion, while the hindgut is responsible for the further processing and absorption of nutrients, as well as the excretion of substances.

## 5. Conclusions

The present study investigated the variations in bacterial taxonomic profiles and potential functions in response to the gut transit of *L. exotica*. Differences in microbial community structure determine the functional specialization of different gut segments in *L. exotica*. The foregut appears to be the primary site of food digestion, while the hindgut is responsible for the further processing and absorption of nutrients, as well as the excretion of substances. The intricate nature of gut microbes renders *L. exotica* better adapted to their complex intertidal ecosystem. These results are references for further resolving the molecular mechanism of the algal preferences of *L. exotica* and understanding their important roles in intertidal ecosystems.

## Figures and Tables

**Table 1:** Statistics table of the top 10 phyla in the foregut and hindgut of *L. exotica*.

Taxonomy	Foregut	Hindgut
Cyanobacteria	46.15%	31.07%
Proteobacteria	26.51%	34.65%
Firmicutes	9.74%	10.05%
Tenericutes	1.91%	6.99%
Planctomycetes	4.31%	4.25%
Actinobacteria	1.72%	4.62%
unidentified_Bacteria	2.58%	3.36%
Bacteroidetes	2.27%	2.23%
Verrucomicrobia	1.07%	1.63%
Acidobacteria	0.79%	0.04%
Others	2.95%	1.12%

**Table 2:** Statistics table of the top 10 families, genera, and species in the foregut and hindgut of *L. exotica*.

Taxonomy	Foregut	Hindgut
unidentified_Xenococcaceae	41.20%	7.50%
*Vagococcus*	1.52%	5.09%
*Pseudoruegeria*	5.27%	0.60%
*Paracoccus*	0.88%	6.12%
*Candidatus_Bacilloplasma*	1.70%	6.92%
*Palleronia*	0.04%	3.57%
unidentified_Bacteria	2.46%	3.35%
*Microbulbifer*	2.37%	1.57%
*Gallicola*	0.89%	1.56%
*Achromobacter*	1.77%	0.21%
Others	41.90%	63.49%

**Table 3:** The diversity index of gut microbial community in the foregut and hindgut of *L. exotica*.

Group	Observed_Species	Chao1	ACE	Simpson	Shannon	Goods_Coverage	PD_Whole_Tree
Foregut	769	778.26	778.029	0.926	6.044	0.999	95.472
Hindgut	757	802.324	811.87	0.863	5.664	0.996	83.866

## Data Availability

The referred to in this study were stored at NCBI’s SRA database as PRJNA1225450 (16S) (https://www.ncbi.nlm.nih.gov/sra/PRJNA1225450, accessed on 14 March 2026), NCBI’s SRA data-base as PRJNA1225498 (metagenome) (https://www.ncbi.nlm.nih.gov/sra/PRJNA1225498, accessed on 14 March 2026) and ProteomeXchange as IPX0011151000 (proteome) (http://proteomecentral.proteomexchange.org/cgi/GetDataset?ID=PXD061000, accessed on 31 May 2026).

## Supplementary Materials

The following supporting information can be downloaded at: https://www.mdpi.com/article/10.3390/microorganisms13040808/s1, Table S1: The proportion of carbohydrate metabolism and amino acid metabolism in the foregut and hindgut based on the KEGG analysis. Table S2: The proportion of gut microbes associated with macromolecular synthesis and catabolism in the foregut and hindgut based on the KEGG analysis. Table S3: The top ten relative abundance statistics table of foregut and hindgut at eggNOG level 2 analysis. Table S4: Table of abundance of resistance genes in the foregut and hindgut of *L. exotica*. Table S5: Differential metabolites in the positive modes. Table S6: Differential metabolites in the negative modes. Table S7: The content of 19 amino acids in the foregut and hindgut. Table S8: The content of common carbohydrates in the foregut and hindgut. Table S9: Statistical table of the content of metabolites significantly more abundant in the hindgut than in the foregut and the content of metabolites present only in the hindgut. Table S10: The concentrations of NO_3_^−^-N and NH_4_^+^-N, along with the elemental percentage of C and N in the foregut and hindgut.
