# Supplementary material for: Differences in Microbial Community Structure Determine the Functional Specialization of Gut Segments of Ligia exotica
Source: Microorganisms. 2025 Apr 2;13(4):808. doi: 10.3390/microorganisms13040808 (PMC12029659; doi:10.3390/microorganisms13040808)
Supplement: Supplementary file 1 [file microorganisms-13-00808-s001.zip › Table S1-S6.pdf]

Table S1 The proportion of carbohydrate metabolism and amino acid metabolism in the foregut and hindgut based on the KEGG analysis.

KO_Pathway_Level3	Hindgut.1	Hindgut.2	Hindgut.3	Foregut.1	Foregut.2	Foregut.3	Description
ko00010	405971	469648.7	535365.8	500522.2	91583.6	83558.847	Metabolism; Carbohydrate metabolism; Glycolysis / Gluconeogenesis
ko00020	197946	410846.3	206492.1	376139.8	56477.1	54763.355	Metabolism; Carbohydrate metabolism; Citrate cycle (TCA cycle)
ko00030	215821	287399.4	287323.8	314510.1	47108.2	42504.701	Metabolism; Carbohydrate metabolism; Pentose phosphate pathway
ko00040	121426	129242	96280.92	145728.5	10555.6	10271.895	Metabolism; Carbohydrate metabolism; Pentose and glucuronate interconversions
ko00051	147528	209528.3	280263.6	237324.2	55224	49604.806	Metabolism; Carbohydrate metabolism; Fructose and mannose metabolism
ko00052	156380	188529.2	324455.6	221447	40397.3	40590.648	Metabolism; Carbohydrate metabolism; Galactose metabolism
ko00053	41652.4	74469.19	35598.9	87811.62	2274.19	2467.5144	Metabolism; Carbohydrate metabolism; Ascorbate and aldarate metabolism
ko00220	136577	246345.7	202654.4	248187.4	44976.6	42572.838	Metabolism; Amino acid metabolism; Arginine biosynthesis
ko00250	342610	477684.8	363626.1	470656.2	95553.3	91930.982	Metabolism; Amino acid metabolism; Alanine, aspartate and glutamate metabolism
ko00260	231141	383451.3	347102.2	418400.4	57667.3	56064.905	Metabolism; Amino acid metabolism; Glycine, serine and threonine metabolism
ko00270	335601	361236.7	460158.7	386212.3	75194.1	72467.829	Metabolism; Amino acid metabolism; Cysteine and methionine metabolism
ko00280	93566.8	426197	115839.9	442596.3	40513.7	39230.224	Metabolism; Amino acid metabolism; Valine, leucine and isoleucine degradation
ko00290	190926	199218.3	183987.5	206256.6	35529.9	33722.146	Metabolism; Amino acid metabolism; Valine, leucine and isoleucine biosynthesis
ko00300	149476	172610.5	219502.4	202478.6	44118.1	42003.938	Metabolism; Amino acid metabolism; Lysine biosynthesis
ko00310	56763.5	244351.2	57198.53	235408.7	228329	228197.4	Metabolism; Amino acid metabolism; Lysine degradation
ko00330	121403	218758	116032.2	236052.5	19307.9	16832.799	Metabolism; Amino acid metabolism; Arginine and proline metabolism
ko00340	64797.6	173726.5	110981.9	188520.5	13373	12730.296	Metabolism; Amino acid metabolism; Histidine metabolism
ko00350	78298.1	150411.6	66616.48	149457.9	29709.8	29853.52	Metabolism; Amino acid metabolism; Tyrosine metabolism
ko00360	66866	215348.6	43348.65	228442.8	8834.61	8454.4422	Metabolism; Amino acid metabolism; Phenylalanine metabolism
ko00380	58924.7	240925.5	61098.95	257043	5440.55	5252.2295	Metabolism; Amino acid metabolism; Tryptophan metabolism
ko00400	135375	199934.9	285474.7	231415.5	54441	50903.769	Metabolism; Amino acid metabolism; Phenylalanine, tyrosine and tryptophan biosynthesis
ko00500	352925	418328.7	555824.4	418553.1	73841.4	70961.641	Metabolism; Carbohydrate metabolism; Starch and sucrose metabolism
ko00520	322887	397050	583650.7	429450	87471.7	84893.452	Metabolism; Carbohydrate metabolism; Amino sugar and nucleotide sugar metabolism
ko00562	55275	80167.95	66949.33	84393.15	4915.88	4664.8511	Metabolism; Carbohydrate metabolism; Inositol phosphate metabolism
ko00620	342158	580418.7	483747.6	597108.7	77122.9	74415.89	Metabolism; Carbohydrate metabolism; Pyruvate metabolism
ko00630	203311	577637.9	215973.6	601283.2	60905.9	60036.764	Metabolism; Carbohydrate metabolism; Glyoxylate and dicarboxylate metabolism
ko00640	192970	353844.8	250773.9	368144.2	66276	62142.555	Metabolism; Carbohydrate metabolism; Propanoate metabolism
ko00650	214729	377704.8	198961.1	412004.4	36236.7	36090.515	Metabolism; Carbohydrate metabolism; Butanoate metabolism
ko00660	116227	126752.8	101931.3	134178.6	21956.4	20558.537	Metabolism; Carbohydrate metabolism; C5-Branched dibasic acid metabolism

Table S2 The proportion of gut microbes associated with macromolecular synthesis and catabolism in the foregut and hindgut based on the KEGG analysis.

KO_ID	Hindgut.1	Hindgut.2	Hindgut.3	Foregut.1	Foregut.2	Foregut.3	Description	
K00516	0	20.124148	0	0	0	0	E1.14.99.55 ǀ lytic starch monooxygenase	Starch
K00703	5451.8633	3737.6976	10669.928	1924.7641	218.04854	243.39978	glgA ǀ starch synthase	
K16147	2438.8803	12698.255	6090.6297	12757.377	91.071687	70.450637	glgE ǀ starch synthase (maltosyl-transferring)	
K21571	473.2428	0	127.36514	0	0	0	susE_F ǀ starch-binding outer membrane protein SusE/F	
K21572	7327.5745	31.779098	3024.9935	7.3845221	2.6205218	2.5094104	susD ǀ starch-binding outer membrane protein, SusD/RagB family	
K21573	4734.6442	37.905707	2221.2733	10.186296	3.6412657	0	susC ǀ TonB-dependent starch-binding outer membrane protein SusC	
K22033	8.1265283	330.89008	26.245131	215.46123	0	0	K22033 ǀ lytic cellulose monooxygenase (C4-dehydrogenating)	Cellulose
K00694	1442.5726	10038.446	7291.1407	3508.3739	23.022772	7.6998559	bcsA ǀ cellulose synthase (UDP-forming)	
K19668	70.189141	1088.4894	35.28195	461.96197	0	0	CBH2, cbhA ǀ cellulose 1,4-beta-cellobiosidase	
K20541	372.61685	490.49917	127.01126	262.68709	5.9646365	8.3674451	bcsB ǀ cellulose synthase operon protein B	
K20543	450.43495	616.04506	114.66044	104.60315	20.354686	49.377871	bcsC ǀ cellulose synthase operon protein C	
K21713	1.6855244	0	0	350.29458	0	0	lpmo ǀ lytic chitin monoxygenase	Chitin
K00698	0	39.642684	1.1177041	41.509742	250.26044	276.49445	CHS1 ǀ chitin synthase	
K01183	7248.8131	6720.8862	2038.524	3887.5432	212.65145	299.12458	E3.2.1.14 ǀ chitinase	
K01452	26.394595	177.42602	0.9155661	3.1525656	0	0	E3.5.1.41 ǀ chitin deacetylase	
K03478	225.51443	3.4744518	172.37188	104.894	2.6139402	9.0374343	chbG ǀ chitin disaccharide deacetylase	
K03791	281.71364	984.15986	102.3725	424.46852	0	0	K03791 ǀ putative chitinase	
K03933	0	149.3438	0	0	0	0	cpbD ǀ chitin-binding protein	
K13381	0	0	31.153403	0	25.300166	16.671798	chiA ǀ bifunctional chitinase/lysozyme [EC:3.2.1.14 3.2.1.17]	
K18454	61.211149	0	0	0	0	0	deaA ǀ chitin disaccharide deacetylase	
K19693	237.04684	0	45.985763	11.75957	0	0	tfoS ǀ AraC family transcriptional regulator, chitin signaling transcriptional activator	
K00688	11290.488	20746.984	34202.466	17636.069	1926.4545	1359.8503	PYG, glgP ǀ glycogen phosphorylase	Glycogen
K00693	0	8.3297756	0	18.521323	70.134106	40.939987	GYS ǀ glycogen synthase	
K00750	0.4853466	3.5587304	0	85.563294	48.285744	13.18907	GYG1, GYG2 ǀ glycogenin	
K01196	0	0	0	9.9332234	259.04702	148.09291	AGL ǀ glycogen debranching enzyme [EC:2.4.1.25 3.2.1.33]	
K02438	563.59273	1894.4643	98.634508	3532.4548	0	0	glgX ǀ glycogen debranching enzyme	
K03083	0	0	9.2762749	0	4.1823043	22.861055	GSK3B ǀ glycogen synthase kinase 3 beta	
K08822	0	0	0	0	17.372649	0	GSK3A ǀ glycogen synthase kinase 3 alpha	
K16150	231.26296	2648.8018	754.44221	4094.1599	3315.3356	3079.9687	K16150 ǀ glycogen synthase	
K06206	906.46818	1533.9384	445.89006	389.73052	21.372916	16.859667	sfsA ǀ sugar fermentation stimulation protein A	Fermentation

Table S3 The top ten relative abundance statistics table of foregut and hindgut at eggNOG level 2 analysis.

Ortholog group description	Foregut	Ortholog group description	Hindgut
Ribonuclease H protein	0.025325	Transcriptional regulator	0.006571
Intron homing	0.025292	ATPase activity	0.005182
Reverse transcriptase (RNA-dependent DNA polymerase)	0.015241	ABC transporter	0.004957
proximal promoter DNA-binding transcription repressor activity	0.01236	rRNA binding	0.004156
Mannose metabolic process	0.012291	Phosphorelay signal transduction system	0.00349
Positive regulation of TOR signaling	0.012005	Transferase activity, transferring glycosyl groups	0.003253
DDE superfamily endonuclease	0.007205	DNA-binding transcription factor activity	0.003221
Transposition	0.006823	Protein conserved in bacteria	0.003148
Transposase and inactivated derivatives	0.006739	Transcriptional regulator	0.003071
Transposition, RNA-mediated	0.006256	Histidine kinase	0.002857

Table S4 Table of abundance of resistance genes in the foregut and hindgut of *L. exotica*.

Resistance genes	Foregut	Resistance genes	Hindgut
vanY_gene_in_vanB_cluster	50.364333	vanY_gene_in_vanG_cluster	133.041333
vanW_gene_in_vanI_cluster	27.39	vanY_gene_in_vanB_cluster	86.358333
vanY_gene_in_vanG_cluster	7.109333	vanT_gene_in_vanG_cluster	74.230333
ErmB	4.618	ErmB	63.879667
tetBP	4.099333	vanW_gene_in_vanI_cluster	47.277667
vanY_gene_in_vanM_cluster	3.993	tetM	46.299667
vanT_gene_in_vanG_cluster	3.867333	vanY_gene_in_vanM_cluster	23.657667
TEM-116	3.833333	tetW	11.512667
tetM	3.771	vanY_gene_in_vanA_cluster	9.687
vanW_gene_in_vanG_cluster	3.626	vanW_gene_in_vanG_cluster	9.645333

Table S5 Differential metabolites in the positive modes.

Name	Fold change	P value	VIP
alpha.-ethyltryptamine	1.849	0.028	3.608
delta.2-cis-eicosenoic acid	1.413	0.035	1.139
(2z)-2-[4-[2-[2-(3,4-dihydroxyphenyl)ethoxy]-2-oxoethyl]-5-methoxycarbonyl-2-[(2s,3r,4s,5s,6r)-3,4,5-trihydroxy-6-(hydroxymethyl)oxan-2-yl]oxy-4h-pyran-3-ylidene]acetic acid	0.311	0.007	1.033
1-(1z-octadecenyl)-2-(5z,8z,11z,14z-eicosatetraenoyl)-sn-glycero-3-phosphocholine	2.854	0.019	2.769
1-(1z-octadecenyl)-2-(5z,8z,11z,14z-eicosatetraenoyl)-sn-glycero-3-phosphoethanolamine	2.825	0.002	2.406
1-(1z-octadecenyl)-2-(9z-octadecenoyl)-sn-glycero-3-phosphocholine	2.462	0.012	2.537
1-hexadecyl-2-(8z,11z,14z-eicosatrienoyl)-sn-glycero-3-phosphocholine	1.869	0.032	1.674
3-methyl-2-buten-1-ol	4.829	0.001	1.318
4-[5-[[4-[5-[acetyl(hydroxy)amino]pentylamino]-4-oxobutanoyl]-hydroxyamino]pentylamino]-4-oxobutanoic acid	2.138	0.010	6.409
4-androsten-17.beta.-ol-3-one glucosiduronate	0.397	0.022	1.068
4-hydroxyisoleucine	1.752	0.028	1.069
5.alpha.-androstan-3.alpha.,17.beta.-diol-o-3-.beta.-glucuronic acid	0.335	0.049	1.021
Adenosine	0.401	0.023	14.400
Arachidoyl ethanolamide	1.369	0.021	1.764
Arctiin	5.989	0.007	1.010
Benzoylnorecgonine	0.502	0.003	1.665
Camphor	0.649	0.049	1.109
Cholecalciferol	2.170	0.007	1.575
Deoxyadenosine	0.309	0.000	7.116
Diacetylpyxinol	7.990	0.047	1.568
Dimethyl sulfone	1.156	0.048	2.150
Ectoine	1.299	0.043	5.235
Keracyanin	0.272	0.039	2.007
Leucylleucine	0.618	0.032	2.991
Lys-Leu	0.504	0.032	1.451
Myristoleic acid	0.368	0.027	4.354
N-(octadecanoyl)sphing-4-enine-1-phosphocholine	3.499	0.032	3.751
N-myristoylsphinganine	1.676	0.003	1.115
Pantethine	3.242	0.050	1.383
Phe-val	0.533	0.017	1.956
Saquinavir	2.806	0.031	1.012
Tetrandrine	2.484	0.034	1.138
Thioetheramide-PC	3.931	0.005	2.174
Thymine	1.890	0.016	4.862
Trp-Leu	0.541	0.041	1.751
Tyr-Phe	0.518	0.020	1.287
Tyramine	1.724	0.013	4.808

Table S6 Differential metabolites in the negative modes.

Name	Fold change	P value	VIP
2-hydroxyatorvastatin lactone	0.315	0.031	1.101
3-(cyclohexylamino)-2-hydroxy-1-propanesulfonic acid	0.534	0.047	2.781
7-benzyl-11,14-dimethyl-16-(2-methylpropyl)-10,13-di(propan-2-yl)-17-oxa-1,5,8,11,14-pent azabicyclo[17.3.0]docosane-2,6,9,12,15,18-hexone	5.484	0.024	1.390
Adenine	0.267	0.024	2.249
Alanine	0.696	0.021	2.437
Andrastin c	12.868	0.020	4.143
Citraconic acid	0.395	0.011	1.157
Corymbosin	0.451	0.034	1.256
D-glucosamine, 6-sulfate	0.285	0.021	1.761
Desferrioxamine d2	22.401	0.020	3.613
His-ser	2.147	0.023	5.565
Linoleic acid	2.465	0.023	8.046
Marticin	2.383	0.044	2.383
Methanone, (4-hydroxyphenyl)(1-pentyl-1h-indol-3-yl)-	0.402	0.005	2.918
Methyl 3,4,5-trimethoxycinnamate	0.438	0.027	1.496
Methylmalonic acid	0.321	0.033	1.467
Muramic acid	0.227	0.029	1.043
N-.alpha.-(tert-butoxycarbonyl)-l-proline	0.448	0.047	1.487
N-octyl sulfate	0.420	0.001	1.498
O-benzyl-d-serine	0.394	0.002	3.071
Porphobilinogen	0.361	0.026	1.021
Pro-gly	0.579	0.023	1.361
Proline	0.429	0.038	10.390
Propanoic acid, 2-[[1-[3-[4-([1,1'-biphenyl]-4-ylcarbonyl)-2-propylphenoxy] propyl]-1,2,3,4-tetrahydro-5-quinolinyl]oxy]-2-methyl-	1.617	0.036	1.091
